# Mitigation of the U.S. agrifood sector’s contribution to human and planetary health: a case study

**DOI:** 10.3389/fnut.2023.1297214

**Published:** 2023-11-16

**Authors:** William H. Dietz, Jessica Fanzo

**Affiliations:** ^1^Global Food Institute, George Washington University, Washington, DC, United States; ^2^Columbia’s Climate School, Columbia University, New York, NY, United States

**Keywords:** climate change, agrifood, beef, political will, policy, communication

## Abstract

The relationship of the United States (U.S.) agrifood sector to climate change is bidirectional; cattle production for beef consumption generates methane and nitrous oxide, both of which are potent greenhouse gases (GHGs). These gases contribute to global warming which in turn increase the frequency and strength of adverse catastrophic events, which compromise the food supply. Increased GHGs also affect crop yields and the micronutrient content of crops, which adversely affect the prevalence of food and nutrition insecurity, particularly in low- and middle-income countries. Because the U.S. is a major contributor to global warming, we have a special responsibility to reduce our contribution to the generation of GHGs. The dilemma is that beef is a highly nutritious and desirable food, with excess consumption in the U.S. and under consumption in other parts of the world, but a desirable source of nutrients in low- and middle-income countries (LMICs). Reductions in fossil fuels have been a major focus of concern, and the agrifood system has been largely ignored. Policy changes to reduce beef consumption have been resisted at the highest levels of government. Furthermore, shifts to more plant-based diets have been contentious. Successful reductions in beef consumption will require individual, institutional, municipal, and state initiatives. Building the political will for change will require a compelling communication campaign that emphasizes the unsustainable contribution of beef consumption to climate change and land and water use.

## The food system and climate change

Climate change and climate-related extreme weather events (droughts, floods, fires, heat, and cold snap spells) adversely impact a whole host of societal systems and the lives that humans are used to (1). Food systems, in particular, are increasingly vulnerable to the effects of climate change. The natural resources – soils, water, biodiversity – and ecosystems essential to producing a wide range of food commodities are threatened or in decline. Extreme weather events have immediate and sometimes devastating consequences on the ability to farm and for farmers and laborers to cultivate food. Longer-term implications of a warmer planet could devastate the ability to grow key crop commodities in the southern latitudes (2). With more carbon dioxide (the main greenhouse gas emitted) in the atmosphere, some micronutrient content of C4 crops will decrease (3). Models suggest that climate change could also spur a phenomenon known as multiple breadbasket failures – in which extreme events could happen simultaneously worldwide, devastating large-scale breadbasket countries meant to feed large swaths of the global population (4).

At the same time, how we manage and govern food systems profoundly impacts the acceleration of climate change and natural resource degradation. In their totality, global food systems generate approximately 30% of total greenhouse gas emissions, much of which comes from the agricultural production of certain commodities with significant environmental footprints (5). While there is disagreement on how much other parts of the food supply chain, such as transport, packaging, and storage, contribute to that total greenhouse gas emission accumulation, it is clear that production and the consumption of foods derived from ruminants (mainly beef and lamb) are significant contributors to global warming (6, 7) with significant variations in those emissions depending on how those foods were grown. Food systems are also heavily dependent on fossil fuels. Producing, trading, moving, and selling food requires significant energy use – from fertilizers to transport to cold chain storage.

While there is a range of foods, including plant-based foods, that have variable environmental footprints across water and land use and greenhouse gas emissions, depending on where they grow, how they are grown and processed, and the practices taken by producers, some groups of foods are more intensive on natural resources and emit more greenhouse gases (8). Cattle production for meat and dairy is the largest emitter of greenhouse gases from the agrifood sector, particularly methane (9). Methane is one of three major greenhouse gases and is one of the most toxic because it traps more heat in the atmosphere than carbon dioxide. In comparing animal source foods, 1 kg of beef from cows generates 99.48 kilograms of carbon dioxide equivalent (kg CO2eq) as compared to 10 kg CO2eq for 1 kg of chicken. Also, 1 kg of soybeans, a high-protein plant-based food, generates 0.8 kg CO2eq.

Those who consume an omnivorous diet have a much higher greenhouse gas footprint than those who consume a plant-rich Mediterranean, vegetarian, or vegan dietary pattern (10). For the world’s agriculture system to produce such a diet, the cattle sector alone would need to contract by 60% (10). The use of the land would also need to be altered significantly. Examining more broadly the use of global land, 40% of the earth’s land is arable, and 77% of that land is used to raise a broad range of animals and the crops to feed them. The remaining 23% of land is used to grow plants. However, animals only generate 18% of the global calories for energy and 37% of calories for protein needs (11). Plants make up the rest. When examining the U.S., cattle (beef) production requires 28 times more land and 11 times more irrigated water compared to poultry, pork and eggs (12). These statistics emphasize that the current use of land and other natural resources is not the most efficient way to grow food for a growing population with significant variations in their environmental intensity depending on the livestock system. Instead, there is significant extensification into biodiversity hotspots.

It is not only the greenhouse gases that are an enormous challenge for the livestock sector. Raising cattle is also a major driver of tropical deforestation (13). This is an issue not only because of the profound and irreversible loss of biodiversity found in forestscapes but also because trees act as a mitigation strategy due to their functionality as carbon sinks (14). Biodiverse-rich sub- and tropical forests such as the Amazon have seen significant deforestation due to agriculture extensification largely due to livestock (and soy).

However, the demand for animal source foods is growing in many parts of the world with income growth. In China, the demand for pork increased from 10 kg per person in 1980 to 45 kg per person in 2022 (15). Brazil and some African countries, such as Ethiopia, are trying to meet that demand by growing their livestock sector. While low- and middle-income countries’ demands are dynamic, there are a set of high-income countries that still consume more meat than is necessary to meet basic nutrient needs, such as the United States, Australia, Brazil, and Argentina, as some examples.

## The need and challenge of reducing beef consumption to mitigate climate change in the U.S.

Because the US is second only to China in the generation of GHGs, and is fourth *per capita* in GHG generation, we bear a moral obligation to lead the way in terms of reducing GHGs. The agrifood sector, with a particular focus on beef consumption, represents one of the most important but neglected target to mitigate climate change. The US agrifood sector generates 10% of GHGs in the U.S. and a total of 85% of those GHGs are generated by cattle production. Cattle produce methane (CH_2_) by enteric digestion of fodder; the overwhelming amount of methane comes from cattle, and almost 75% of that methane comes from beef cattle; the remainder comes from dairy cattle (16). Methane is approximately 80 times more powerful than CO_2_ but has a relatively short atmospheric half-life. An additional source of GHG production related to cattle production comes from the fertilizer used to grow the fodder consumed by cattle. Fertilizer that is not used by plants is converted to nitrous oxide (N_2_O), a GHG that is 265 times more powerful than CO_2_, and has a long atmospheric half-life. In terms of their contribution to GHGs, nitrous oxide emissions are roughly equivalent to methane emissions (16).

Meat production and consumption go hand in hand with human and planetary health on an acute and chronic basis. Increased GHGs contribute to catastrophic weather events that immediately affect the food supply. On a longer term basis, increased GHGs reduce crop yields. Together with decreased crop yields, the decreased micronutrient of food causes food and nutrition insecurity, and increased beef consumption contributes to cardiovascular disease, colon cancer, diabetes, and obesity (17–19). Together, these interactions contribute to the global syndemic, but also point to the possibility of triple duty solutions that promote human and planetary health.

A recent study examined the environmental and health impact of four dietary indices based on the alternative healthy eating index 2010 (HEI-2010) (18). Higher (healthier) AHEI-2010 scores were associated with a decreased risk of cardiovascular disease (CVD) and a lower environmental impact. Red and processed meat was the biggest factor affecting both the AHEI score and more adverse environmental impacts. Beef consumption also was the biggest contributor to GHGs, cropland use (59%), irrigation water (26%) and fertilizer (8.5%) (18). As shown in the Table 1, as beef consumption decreases and consumption of more plant-based diets increases, GHGs, land and water use, and biodiversity improve (20). Even a modest 10% decrease in beef consumption will have positive effects (21).

**Table 1:** Environmental impact of dietary choices.

Group	CH_4_ Kg/d	N_2_O Kg/d	Land use m^2^/d	H_2_O use m^3^/d
Vegans	4.4	0.7	4.4	0.4
Vegetarians	20.	1.0	6.0	0.5
Low meat-eaters (28g/d)	29.0	1.3	8.3	0.7
Medium meat eaters (50–99 g/d)	40.8	1.7	11.3	0.8
High meat eaters (140g/d)	65.4	2.6	16.8	0.9

Decreased meat consumption and increased plant-based diets are associated with reduced GHG emissions, and land and water use. Adapted from Scarborough et al. (20).

The challenge is how to reduce meat intake. In the U.S., Men consume more beef/capita than women (86 vs. 48 lbs./capita/y), and ground beef (burgers) constitutes 42% of beef consumed (22). Consumption has somewhat decreased recently, but the sex dichotomy has persisted. Twenty eight percent of ground beef is consumed at restaurants, and most of the restaurants are likely fast food restaurants.

Despite the beneficial effects of reducing beef consumption, its importance as a target for mitigating climate change has largely been ignored. For example, the 2022 Policy Brief for the United States of America – Lancet Countdown on Health and Climate Change (23) failed to acknowledge the importance of the agrifood system and offered no strategies to reduce beef production. Furthermore, the role of beef production has received only limited attention from mainstream media. In a survey of 1,000 articles related to the causes of climate change in ten major media sources, such as the Wall Street Journal or the New York Times, animal agriculture was cited in only 7% of articles as a contributor to climate change (24). Therefore, it is not a surprise that only 3% of US consumers rank industrial meat compared to 21% of US consumers that rank fossil fuels as the major contributor.

## Federal responses to efforts to reduce beef consumption in the U.S.

The U.S. Dietary Guidelines for Americans (DGAs) provide the most comprehensive nutritional recommendations for federal programs and the general public. However, efforts to address beef consumption in the context of sustainability have met resistance at the highest levels of government. The most egregious example occurred in response to the recommendations of the 2015–2020 DGA Advisory Committee. One of their recommendations was that sustainability, which clearly included reductions in beef consumption, be considered in the DGAs (25). In response, the meat industry conducted a vigorous and successful lobbying effort that prompted the Secretaries of Health and Human Services and the Department of Agriculture to announce that sustainability would not be included as a DGA criterion (26). That stance has continued with the 2020–2025 DGAs.

The response of the Trump administration to the closure of meat packing plants during the COVID-19 pandemic provides another example of the power and politicization of the beef industry. In response to packing plant closures, President Trump declared that packing plants for beef and poultry were “critical infrastructure” (27) and issued an Executive Order declaring that operations in these packing plants continue, despite the high rates of Covid-19 infections and deaths among meat packing workers (28, 29). In effect, disruptions of the beef supply chain were considered a national emergency.

The absence of policies to reduce beef consumption have not fared much better under the Biden-Harris administration. In 2022, the report of the White House Conference on Hunger Nutrition and Health made only one reference to climate change, and that focused on research rather than actionable strategies to increase the consumption of sustainable foods (30).

A number of federal policy initiatives for the reduction of beef consumption have been proposed (31, 32). These include strengthening dietary guidelines, taxes on GHG emissions, removal of agricultural subsidies that maintain low beef prices, and communication campaigns. Federal policy changes in the U.S. are unlikely, given the vocal but influential minority that denies the existence of climate change and refuses to support changes that mitigate it. Suggestions to reduce beef consumption are met with similarly polarized attitudes in the public domain that split along all or nothing lines – either vegan or vegetarian diets versus beef consumers. The latter argue that the adverse effects of beef on health lack scientific evidence, impair individual freedom, and characterize vegans of plotting a near vegan diet for the world’s population (31). Resistance to policy changes directed at reducing beef consumption are characterized by highly polarized responses. For example, the “war on meat” has been described as “the devil is a shapeshifter…he takes the form of demonic foods. In response the armies of the righteous have already waged war on sugar, and now red meat is in their sights.”

## The need for local strategies to build political will in the U.S.

These observations emphasize the need to move from a focus on federal policy to one which builds on individual, institutional, municipal and state policy to generate political will from the ground up. Increased awareness of the adverse effects of beef consumption on human and planetary health can lead to changes at the individual level that extend to family and friends. At the institutional level, procurement policies, like those based on the federal food service guidelines, can be used to reduce the purchases of beef and increase the availability of plant-based options. A default strategy, which made plant-based main dishes the default option in cafeterias effectively changed food choices in university settings (33).

Effective communication efforts will be essential. These efforts should emphasize that the nutritional benefits of beef in the provision of protein, iron, zinc and vitamin B_12_ can readily be achieved at levels of intake below the current excess intakes that are consumed. Significant efforts will be required to identify the most cogent arguments that appeal to men. Communication strategies need to be adequately tested but could include the following (31).

- Focus on reduction, not elimination
- Acknowledge the positive health effects of beef consumption in HICs and LMICs
- Emphasize that beef consumption in North America and Eurasia exceed recommended consumption by 6 and 3 times, respectively (6)
- Present the case that both planetary and human health are adversely affected by beef production and consumption
- Emphasize the effects that reduced beef consumption/production will have on land, water, fertiliser, and GHGs
- Provide compelling examples: GHGs from 1 serving beef = GHGs from 20 servings of vegetables; land that produces 100gm plant protein produces only 4 gm beef protein

Two relevant experimental studies have assessed the impact of messaging on discouraging red meat consumption. An online study compared the impact of messages related to animal welfare, health or the effects of red meat production on climate change with a neutral non-red meat control in a survey of 2,773 non-vegetarian and non-vegan adults. Adults who received the message regarding the effects of red meat consumption on climate change were significantly more likely to indicate that they would reduce their red meat consumption at full service restaurants than those who received the messages about health and animal welfare (34). A second study of college students found that students ranked reduced meat intake as less effective than other measures to address climate change, such as recycling and using less plastic. However, among students who reported that making food choices that were good for the environment, consuming foods that reduced climate impact, or that eating less red meat was an effective way to reduce climate change reported a 10%–25% lower frequency of red meat consumption (35).

## The dilemma

Reductions in beef consumption pose a dilemma. In the U.S., beef is a highly desirable and valued food that is over-consumed compared to nutritional requirements (6). Beef is also a rich source of protein, vitamin B12, iron and folic acid lacking in the diets of the global south, making it a valuable source of nutrients. The dilemma is how to reduce beef consumption in the U.S. to reduce climate change and simultaneously increase beef consumption in lower- and middle- income countries without increasing GHG production. One of the unanticipated adverse consequences of reduction in beef consumption in the US is that beef exports could increase without a reduction in production. This possibility emphasizes the need for global efforts to achieve an overall reduction in beef consumption while achieving a redistribution that meets the nutrient needs of LMICs.

## Summary

The need to reduce GHGs is urgent. Fifteen percent of fossil fuel use is attributable to the transportation sector, most of which is attributable to car use. The agrifood system generates 10% of GHGs, 85% of which is attributable to the production and consumption of meat. The product of GHGs from fossil fuels is CO_2_ which has a half-life of over 100 years, whereas the GHG products of beef production are methane and nitrous oxide. Methane is 80 times more powerful than CO_2_ and its half life is approximately 10 years. Therefore, reductions in meat consumption and their consequent effect on meat production promises a much more rapid effect on GHGs. The challenge is how to reduce beef consumption in the U.S. We suggest that federal policy initiatives are unlikely to succeed given the polarization in Congress, and that change needs to start with individuals, their families, social networks, and institutions, and municipalities to generate the political will necessary to accomplish reductions in beef consumption. An effective communication strategy will be essential. Rapid change is essential for the health of humans and the planet.

## Data availability statement

The original contributions presented in the study are included in the article/supplementary material, further inquiries can be directed to the corresponding author.

## Author contributions

WD: Conceptualization, Writing – original draft, Writing – review & editing. JF: Conceptualization, Writing – original draft, Writing – review & editing.
